# Severe falciparum malaria treated with artesunate complicated by delayed onset haemolysis and acute kidney injury

**DOI:** 10.1186/s12936-015-0760-x

**Published:** 2015-06-18

**Authors:** Katherine Plewes, Md Shafiul Haider, Hugh W F Kingston, Tsin W Yeo, Aniruddha Ghose, Md Amir Hossain, Arjen M Dondorp, Gareth D H Turner, Nicholas M Anstey

**Affiliations:** Mahidol Oxford Tropical Medicine Research Unit, Faculty of Tropical Medicine, Mahidol University, Bangkok, Thailand; Nuffield Department of Medicine, Centre for Tropical Medicine and Global Health, University of Oxford, Oxford, UK; Division of Infectious Diseases, Department of Medicine, University of British Columbia Clinical Investigator Program, Vancouver, Canada; Department of Medicine, Chittagong Medical College Hospital, Chittagong, Bangladesh; Global Health Division, Menzies School of Health Research and Charles Darwin University, Darwin, NT Australia

**Keywords:** Falciparum malaria, Delayed haemolysis, Acute kidney injury

## Abstract

**Background:**

Severe falciparum malaria may be complicated by haemolysis after parasite clearance, however the mechanisms remain unclear. Recent reports describe a pattern of delayed onset haemolysis among non-immune travellers with hyperparasitaemia treated with intravenous artesunate, termed post-artesunate delayed haemolysis (PADH). The occurrence and clinical impact of PADH following severe malaria infections in areas of unstable transmission are unknown.

**Case:**

A 45-year-old Bangladeshi male was initially admitted to a local hospital with severe falciparum malaria complicated by hyperparasitaemia and treated with intravenous artesunate. Twenty days from his first presentation he was readmitted with delayed onset haemolytic anaemia and acute kidney injury. Multiple blood transfusions and haemodialysis were required. Renal biopsy revealed acute tubular injury and haem pigment nephropathy. His haemoglobin and renal function recovered to baseline after 62 days from his second admission.

**Discussion:**

This case highlights the differential diagnosis of post-malaria delayed onset haemolysis, including the recently described syndrome of post-artemisinin delayed haemolysis. The pathophysiology contributing to acute kidney injury in this patient and the limited treatment options are discussed.

**Conclusions:**

This report describes PADH complicated by acute kidney injury in an adult patient living in a malaria hypoendemic region who subsequently required blood transfusions and haemodialysis. This case emphasizes the importance of routine follow up of haemoglobin and renal function in artesunate-treated patients who have recovered from severe malaria.

## Background

Delayed onset haemolytic anaemia is a recognized but infrequent complication during recovery after severe falciparum malaria infection. The course of anaemia following severe malaria is often protracted and, in those treated with quinine, haemoglobin may not return to baseline until 28 days after infection [1]. Prior to the introduction of artesunate, prolonged haemolysis contributed to this delayed haemoglobin recovery in less than 7% of patients [1]. The mechanisms of continued haemolysis after parasite clearance are unclear, with both auto-immune and non-immune processes implicated in the absence of artesunate treatment [2–4]. Recent reports describe a syndrome termed post-artemisinin delayed haemolysis (PADH) following treatment with intravenous artesunate [5–11]. PADH usually occurs 1–3 weeks after artesunate is administered to non-immune travellers with high parasitaemia. The different patterns of haemolysis following malaria infection and artesunate treatment highlight the complexity of malaria anaemia.

Massive haemolysis is frequently associated with haemoglobinuria and acute kidney injury (AKI) [12–15]. When haemoglobin and haem scavengers, haptoglobin and haemopexin, are saturated the haemoglobin is filtered through the glomerulus. It is then re-absorbed and catabolized in the proximal tubule. Haemoglobinuria results when re-absorption capacity is reached [16]. Studies implicate cell-free haemoglobin in the mechanism of AKI in haemolytic conditions [17, 18]. This report describes the first case of a patient from an unstable malaria transmission area presenting with delayed-onset haemolysis complicated by acute kidney injury following treatment of severe malaria with intravenous artesunate and follow-on oral artemether lumefantrine combination treatment.

## Case presentation

A 45-year-old, previously well, Bangladeshi male presented to Chittagong Medical College Hospital with a 4-day history of fever, headache, prostration, myalgias, nausea, and anorexia. This center is the main referral hospital for the Chittagong Division in southern Bangladesh, which is endemic for malaria with unstable transmission. One day prior he was diagnosed with malaria based on a positive rapid diagnostic test (RDT).

There was no significant past medical history, specifically no prior malaria infections, chronic illness, jaundice, liver or kidney disease, transfusions, malignancy, or auto-immune disease. There was no history of passing red or black urine. He was not taking any regular prescription or traditional medications and had no prior exposure to artemisinins.

On admission (day 0), his temperature was 40.3°C, Glasgow Coma Score (GCS) 15/15, blood pressure (BP) 117/62 mmHg; heart rate (HR) 102 beats per minute (bpm) and respiratory rate 36 breaths per minute with 98% oxygen saturation. He appeared well with mild dehydration and scleral icterus, but otherwise unremarkable examination. He was diagnosed with uncomplicated malaria on admission and received one dose of oral artemether/lumefantrine combination (AL) 80/480 mg (Novartis) since he had no clinical criteria for severe malaria, no initial blood tests and only an RDT-positive test.

Subsequent blood tests confirmed a diagnosis of severe falciparum malaria based on hyperparasitaemia (13.8% erythrocytes infected; 659,000 parasites/μL) and hyperbilirubinaemia (bilirubin 80 μmol/L). He was promptly switched to intravenous artesunate 120 mg, 2.4 mg/kg body weight, (Guilin No. 2, Pharmaceuticals, China) and received three doses at 12-h intervals. He was also randomized to the normal saline arm of a randomized controlled trial of adjunctive l-arginine in severe malaria (ACTRN12612000571875). After 24 h he defervesced and was switched to oral AL for 3 days (total of 480 mg artemether and 2,880 mg lumefantrine). His recovery was unremarkable except for a delayed parasite clearance (estimated half life 8.2 h). Due to this persistent parasitaemia, he received a second 3-day course of AL and his parasites cleared at 148 h. In total he received 13 doses of AL (total dose of 1,040 mg of artemether and 6,240 mg of lumefantrine) and three doses of intravenous artesunate (total dose of 360 mg). He was discharged 8 days later with full recovery, a stable haematocrit and creatinine at a baseline of 86 μmol/L.

At follow-up on day 14 after admission he was asymptomatic, however his white blood count revealed a significant leukocytosis and severe normocytic anaemia (haemoglobin of 60 g/L; Table 1). He declined re-admission and subsequently experienced fever, rigors, headache, jaundice, abdominal pain, and vomiting. He had not taken any antibiotics, non-steroidal anti-inflammatories or proton pump inhibitors during this intercurrent period after treatment of the initial malaria episode.

**Table 1:** Investigations

Parameter	Day 0	Day 3	Day 14	Day 20	Day 44	Normal range
Plasma haemoglobin (ng/mL)^a^	177,839	–	–	127,146	–	<124,000
Haemoglobin (g/L)	141	136	60	49	102	140–180
Haematocrit (%)	43.6	40	18.7	16	27.1	47–54
Mean corpuscule volume (fL)	86.3	–	86.2	94.7	85.2	76–96
Plasma haptoglobin (μg/mL)^a^	1,160	–	–	5	–	300–2,000
Platelets (×10^3^/μL)	28	–	228	478	320	150–00
White blood cells (×10^3^/μL)	6.23	–	19.9	9.8	10.5	4–10
Neutrophils (%)	83		–	70	60	40–70
Creatinine (μmol/L)	97	86	–	1,769	132	53–115
Potassium (μmol/L)	3.7	4.4	–	8.4	4.8	3.5–4.9
Venous pH	7.41	–	–	7.2	–	7.31–7.41
Venous bicarbonate (mmol/L)	19.5	–	–	8.7	–	23–28
Venous carbon dioxide (mmHg)	31.2	–	–	23.5	–	41–51
Venous base excess (mmol/L)	−5	–	–	−20	–	(−2) to (+3)
Venous lactate (mmol/L)	2.36	–	–	0.58	–	0.9–1.7
Anion gap (mmol/L)	13	–	–	6	–	10–20
Total bilirubin (μmol/L)	80	–	–	–	–	3–21
Indirect bilirubin (μmol/L)	26	–	–	–	–	2–14
Lactate dehydrogenase (U/L)	592	–	–	887	–	140–280
International normalization ratio	–	1.3	–	–	–	0.8–1.2
Partial thromboplastin time (seconds)	–	15	–	–	–	<30

^a^Specialized tests performed on samples sent to Darwin, Australia.

**Figure 1:**
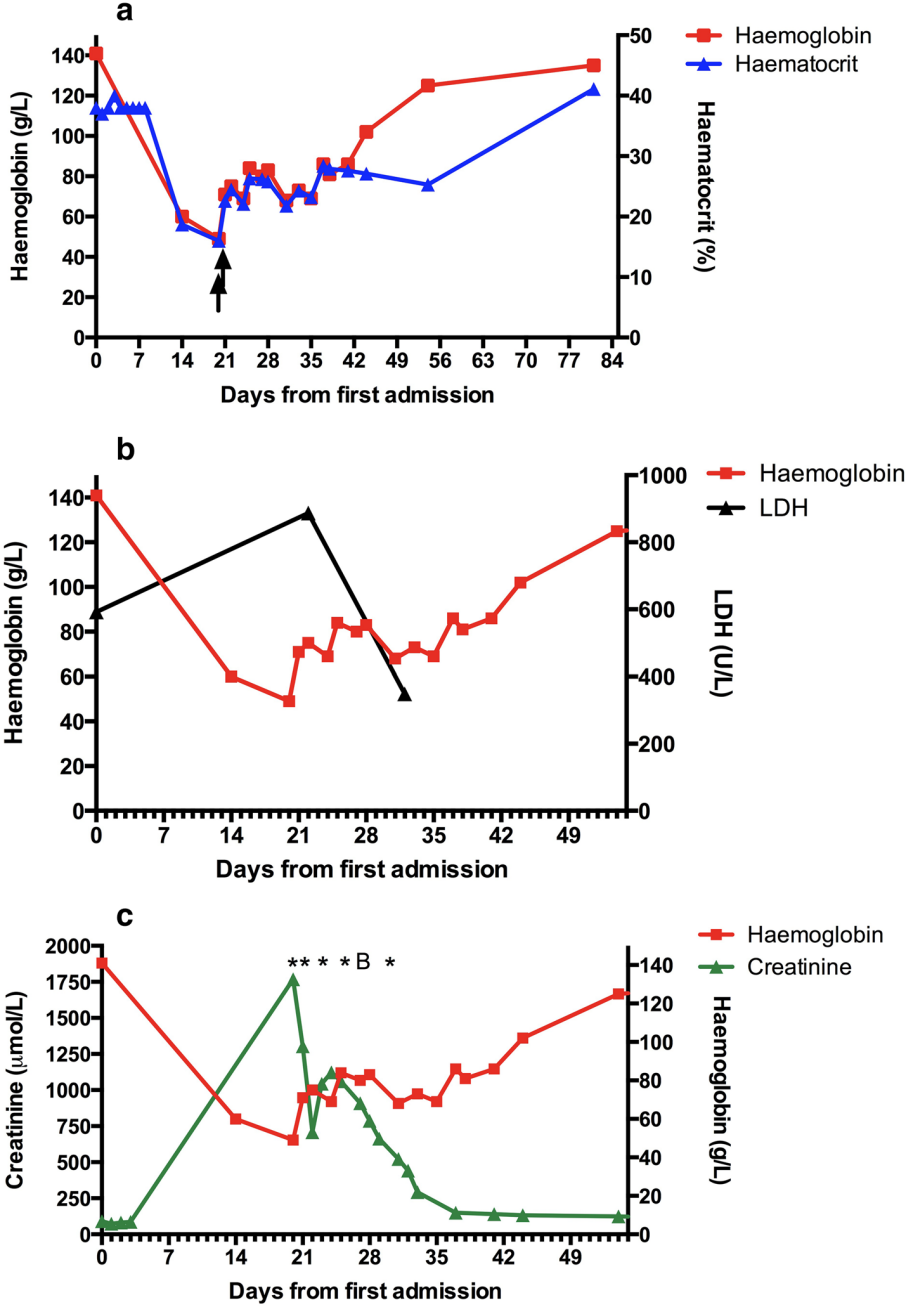
Haemoglobin, haematocrit, creatinine, and lactate dehydrogenase profiles from both hospital admissions. The first admission for severe malaria with hyperparasitaemia and hyperbilirubinaemia from day 0 to day 8; second hospital admission for severe haemolytic anaemia and acute kidney injury from day 20 to day 44. **a** Haemoglobin and haematocrit profile during hospital admissions. Haematocrit was stable during first hospital admission. Haemoglobin dropped by 57 and 65% on days 14 and 20, respectively, and recovered by day 81. *Arrows* indicate timing of whole blood transfusions on days 20 and 21; **b** haemoglobin and lactate dehydrogenase (LDH) profile during hospital admissions; **c** haemoglobin and creatinine profile during hospital admissions. Acute kidney injury, with a creatinine increase by 21× on second hospital admission, classified as stage 3 according to AKIN criteria and ‘failure’ by RIFLE criteria. Presumed baseline creatinine from day 1 was 74 μmol/L. His creatinine returned close to baseline by day 81 (106 μmol/L). *Asterisks* indicate haemodialysis on days 20, 21, 23, 25, and 30; ‘*B*’ indicates renal biopsy on day 27. *AKIN* acute kidney injury network, *RIFLE* risk, injury, failure, loss, end-stage kidney disease.

He was re-admitted on day 20 feeling unwell with generalized oedema, particularly of the face and lower extremities. Vital signs were stable: temperature 36.5°C, GCS 15, BP 130/74 mmHg and HR 78 bpm. He appeared sick, anaemic and had periorbital oedema. Cardiac examination revealed a systolic flow murmur at the left upper sternal border with no radiation. There was mild abdominal tenderness on palpation and bilateral lower limb pitting oedema. Biochemistry revealed AKI with a creatinine increased to 1,769 μmol/L, hyperkalaemia, a non-anion gap hyperchloraemic metabolic acidosis and severe anaemia with 4+ haemoglobinuria (Table 1), with other investigations outlined below. Bladder catheterization did not suggest urinary retention with a residual volume of less than 50 mL of dark orange urine. He promptly received calcium gluconate and insulin for hyperkalaemia, empiric ceftriaxone, and was referred for haemodialysis and blood transfusion. Following five rounds of haemodialysis and three units whole blood transfusion, his metabolic acidosis, hyperkalaemia, renal function, and anaemia improved (Figure 1).

He was discharged recovering well on day 44 with stable serum creatinine and haemoglobin. He was followed up weekly thereafter, and by day 81 his kidney function improved further towards baseline (creatinine 115 μmol/L), and his haemoglobin recovered (135 g/L).

## Investigations

Investigations are summarized in Table 1. Samples sent to Thailand and Australia for specialized tests are indicated by an asterisk. On day 20, reticulocytes accounted for 3% of erythrocytes (reticulocyte production index 0.4). The direct antiglobulin test (DAT; anti-IgG and anti-C3d) and indirect antiglobulin test (IAT) were negative. Plasma cell-free haemoglobin* was elevated (127,000 ng/mL; normal range <124,000 ng/mL), and haptoglobin* decreased (5 μg/mL; normal range: 300–2,000 μg/mL). A 24-h urine collection revealed 525 mg of protein (normal range <150 mg/day). Urine microscopy showed red blood cells casts and dysmorphic red cells. Urine biochemistry*: osmolarity 283 mOsm/kg, sodium 84 mmol/L, urine anion gap of 8, urine osmolar gap 21, and transtubular potassium gradient 1.8. Anti-nuclear antibodies were absent and C3/C4 levels were normal. HIV and hepatitis C antibodies, hepatitis B surface and envelope antigen were negative. Blood cultures and bacterial 16S* ribosomal DNA amplification performed to exclude bacteraemia as a cause of the leukocytosis were negative. Repeat peripheral blood smears for malaria were negative and no schistocytes were found. Glucose-6-phosphate dehydrogenase (G6PD) was qualitatively normal on day 0.

Abdominal ultrasound on day 22 showed normal kidney size with increased parenchymal echogenicity and poor corticomedullary differentiation, with no pelvicalyceal system dilatation.

**Figure 2:**
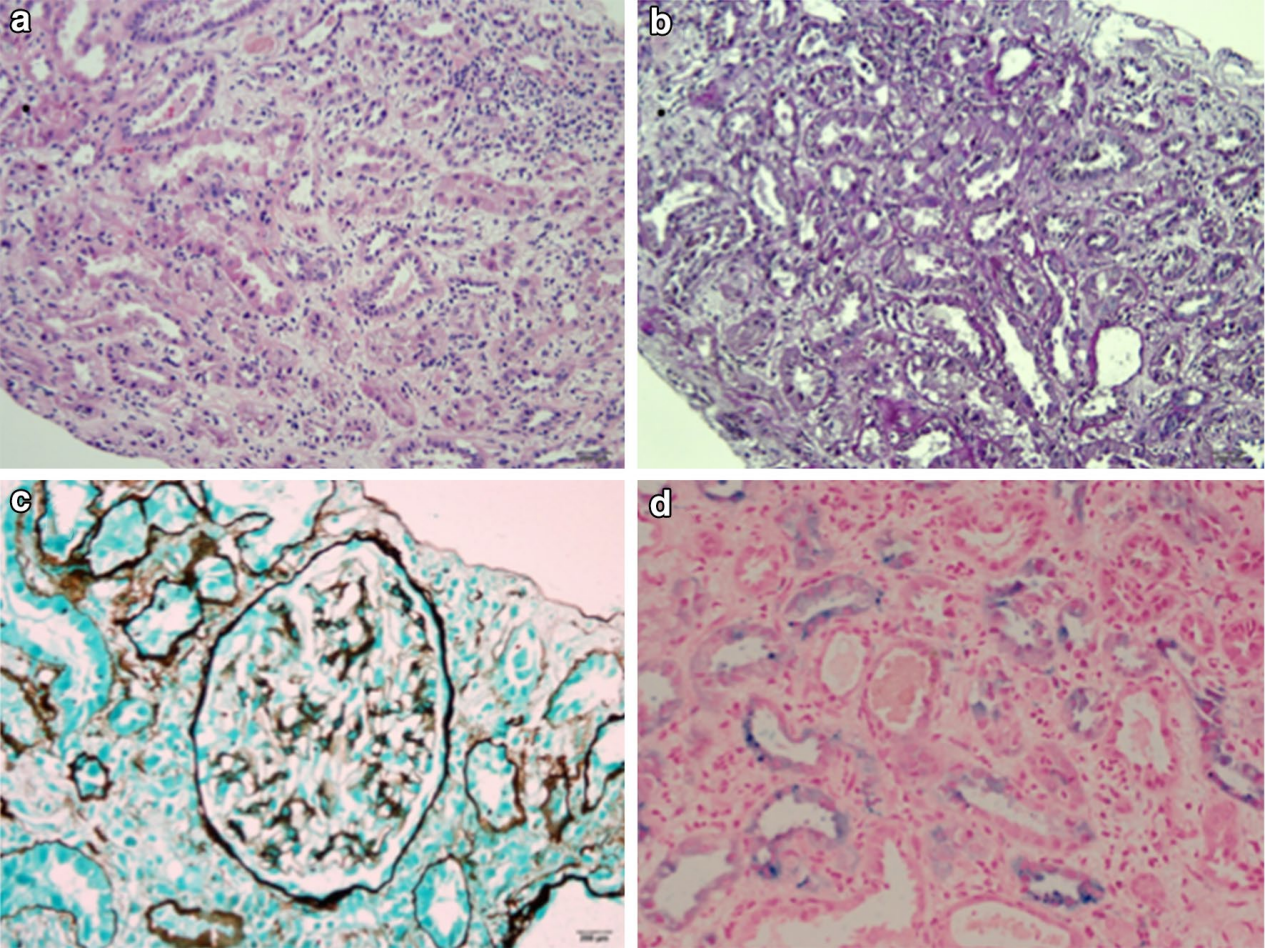
Renal biopsy. Left kidney ultrasound guided biopsy performed on day 27. Photomicrographs to show histopathological findings; **a** acute tubular necrosis with dilation, epithelial thinning, tubular cell necrosis and regeneration, interstitial oedema, and minimal inflammation (haematoxylin and eosin stain ×200); **b** acute tubular injury with PAS staining showing loss of brush border in the dilated injured proximal tubules (Periodic acid Schiff ×200); **c** a normal glomerulus (×400 Jones methanamine silver stain), and **d** a Perls stain for iron showing particulate and diffuse staining in damaged proximal tubules (×400). Specialized PAS, methanamine, and Perls straining were performed in Bangkok, Thailand since only haematoxylin and eosin stain was available in Bangladesh.

A renal biopsy performed on day 27 revealed severe and recovering acute tubular injury, with interstitial oedema but only minimal tubulointerstitial inflammation (Figure 2). Tubules showed acute tubular dilatation, injury and regenerative features. Several red cell casts and amorphous casts were present. Vessels were normal. Glomeruli were morphologically normal with no active glomerulonephritis, negative immunoperoxidase staining for immunoglobulins and C3, and no immune complex deposition or basement membrane abnormalities on electron microscopy. A myoglobin immunostain was negative in casts. However, a Perl’s stain for iron showed both diffuse tubular staining and increased deposition in granular deposits within the cytoplasm of damaged tubular epithelial cells.

## Discussion

This report describes a case of post-artesunate delayed-onset haemolysis complicated by acute kidney injury after recovery from severe falciparum malaria with hyperparasitaemia. Identifying the cause of haemolytic anaemia following malaria infection is challenging, as its mechanism is multifactorial and the broad differential diagnosis requires specialized testing that is often unavailable in resource-limited settings. The timing of haemolysis in this case 14 days after the first dose of artesunate accompanied by a >10% drop in haemoglobin (from day 7 haemoglobin), haptoglobin <0.1 g/L and lactate dehydrogenase >390 U/L is consistent with the case definition of PADH [5, 7, 8]. Since 2012, PADH is proposed to have occurred in at least 38 patients [19, 20], however these reports do not strictly adhere to the optimized definition of PADH recently published in 2014 [5, 7]. Given this limitation it is difficult to accurately determine the frequency and clinical impacts of PADH. A recent review of the reported cases with an alternative PADH case definition estimates that among severe malaria patients treated with intravenous artesunate PADH occurs in 13% and blood transfusions are required in 9% [20]. Of these PADH cases, 31 were non-immune returning travellers, six were immune African children under 5 years, and one was an immune West African adult [6, 9–11, 20–26]. The case reported here describes PADH in a non-immune adult Bangladeshi patient who recovered from an episode of severe malaria acquired while living in an unstable malaria transmission region.

The mechanism of PADH is hypothesized to be due to pitting of ring staged parasitized red cells, where the spleen removes the dead parasite from the red cell akin to the removal of Howell–Jolly bodies [5]. These pitted cells are formed quickly after treatment with artesunate and have a shortened red cell survival [27–29]. Destruction of the once-infected pitted cells manifests as delayed onset of haemolysis. However, in this case and other reported cases of PADH [30] the magnitude of the drop in haemoglobin exceeds the fall expected if only the once-infected pitted cells are destroyed. This suggests that additional artesunate-associated mechanisms of delayed haemolysis involving uninfected red cells must occur. Inhibition of erythropoiesis has also been proposed to contribute to the anaemia as it has been suggested that patients receiving higher doses of artesunate are at higher risk of bone marrow suppression [7]. This may have contributed to the anaemia in the presented case as he received a total dose of 7.2 mg/kg artesunate and 20.8 mg/kg artemether, and a day 20 reticulocyte production index of <2% supported an inadequate bone marrow response to his haemolysis. The young ring-stage activity of artesunate is one characteristic that confers its life-saving benefit over quinine. Artesunate remains first-line anti-malarial for the treatment of severe malaria since the benefit of survival outweighs the low probability of non-life-threatening delayed haemolysis. Recent guidelines recommend weekly monitoring malaria patients for indicators of haemolysis for 1 month following treatment with artesunate [30].

This case of delayed haemolysis was complicated by severe AKI, which has not been reported in the setting of PADH. The presence of excess haem pigment in tubular cells may be due to re-absorption from tubular red cells or haemoglobinuria. The role of haemoglobinuria has been associated with AKI in a spectrum of diseases including blackwater fever, babesiosis, paroxysmal nocturnal haemoglobinuria, massive transfusion and post-cardiopulmonary bypass [12–15, 17, 18, 31]. While not all cases of haemoglobinuria result in AKI, up to 64% of patients with blackwater fever develop renal dysfunction [14]. In this case the features suggested direct tubular epithelial toxicity due to free haem rather than a cast nephropathy. The pathophysiology of haem-pigment AKI is not completely defined but vasoconstriction; direct tubular cell injury and tubular obstruction are thought to contribute [32]. There is accumulating evidence that haemoproteins induce oxidative renal damage by lipid peroxidation [17, 33]. The redox cycling of the haem group initiates lipid peroxidation, releasing potent oxidative compounds that cause renal vasoconstriction [33]. This mechanism is demonstrated in post-operative AKI after post-cardiopulmonary bypass where high cell-free haemoglobin and oxidative stress were associated with kidney injury [17, 18]. Cell-free haemoglobin is also likely to cause vasoconstriction through its quenching of endothelial nitric oxide (NO) in severe falciparum malaria, where it has been associated with impairment in NO-dependent endothelial function [34]. The renal pathology in this case is consistent with haem-pigment induced AKI with clinical evidence of non-anion gap hyperchloraemic metabolic acidosis with distal tubule dysfunction likely secondary to hypovolaemia and vasoconstriction.

The differential diagnosis for haemolysis in this case is broad and the possible contribution of an intercurrent bacterial infection between admissions was considered given his leukocytosis on day 14. Infections may cause haemolysis by precipitating oxidative stress in patients with underlying red blood cell (RBC) disorders, inducing haemolytic pathologic responses, or from the antimicrobial therapy itself [35]. Limited diagnostics suggested no underlying RBC abnormality: normal qualitative G6PD, mean corpuscular volume (MCV) and peripheral smear. Negative DAT and IAT argued against an immune-mediated haemolytic anaemia. Infections characterized by haemolysis include: malaria, babesiosis, Carrion’s disease, and typhoid. The peripheral smear on second admission revealed no intra-erythrocytic organisms. Negative blood cultures and bacterial 16S ribosomal DNA amplification were not conclusive but argued against infection as a cause of the haemolysis. Furthermore, the leukocytosis observed on day 14 had resolved by day 20 in the absence of antibiotic treatment.

The differential diagnosis for the etiology of AKI included (1) pre-renal, and (2) intrinsic renal causes including: acute tubular injury (ATI) due to ischaemia or direct toxin-mediated injury by cell-free haemoglobin, acute interstitial nephritis (AIN) secondary to β-lactams, microangiopathy, or post-infectious glomerulonephritis (PIGN). The urinary osmolarity <350 mOsm/kg and fractional excretion of sodium >1% did not suggest a pre-renal cause. The absence of systemic eosinophils and eosinophil-rich tubulo-interstitial inflammation on renal biopsy argued against a diagnosis of AIN, although recovering AIN can be pauci-inflammatory. The normal platelets, lack of schistocytes, negative blood cultures and 16S rRNA amplification, and normal renal vessels and glomeruli on biopsy were not consistent with haemolytic uraemia syndrome/thrombotic thrombocytopenic purpura or PIGN.

Treatment for established AKI is limited to renal replacement therapy. Research towards prevention and alternative treatments is vital since dialysis is frequently unavailable. Currently, there is no targeted treatment for cell-free haemoglobin-induced AKI, however results from experimental models and case series are encouraging. Recent studies have shown that paracetamol inhibits haemoprotein-induced lipid peroxidation, resulting in decreased oxidative kidney injury and improved renal function [36, 37]. Administration of haptoglobin infusions is shown to attenuate cell-free haemoglobin-mediated AKI in paroxysmal nocturnal haemoglobinuria and an experimental model of stored blood transfusion [38, 39].

## Conclusions

Malaria treatment guidelines should consider recommending that renal function be checked if a drop in haemoglobin is detected during monitoring of severe malaria patients after treatment with artesunate. This may be logistically difficult in limited-resource countries but where it can be done, monitoring the creatinine should be a part of severe malaria management to detect and avoid renal complications.

### Learning points

- Delayed onset haemolytic anaemia following parasite clearance in severe malaria infection has a broad differential diagnosis to consider.
- Delayed onset haemolysis following severe malaria may occur following artesunate treatment, and be complicated by AKI.
- Severe malaria patients treated with artesunate should be monitored weekly for decline of haemoglobin and renal function.
- There are limited treatment options to treat haemoprotein-mediated AKI.

## Abbreviations

AIN: acute interstitial nephritis
AKI: acute kidney injury
AL: artemether lumefantrine
ATI: acute tubular injury
BP: blood pressure
bpm: beats per minute
DAT: direct antiglobulin test
G6PD: glucose-6-phosphate dehydrogenase
GCS: Glasgow coma score
HIV: human immunodeficiency virus
HR: heart rate
IAT: indirect antiglobulin test
MCV: mean corpuscular volume
NO: nitric oxide
PADH: post-artesunate delayed haemolysis
PIGN: post-infectious glomerulonephritis
RBC: red blood cell
RDT: rapid diagnostic test
